# Outcomes of robotic modified Freyer's prostatectomy in an Australian patient cohort

**DOI:** 10.1002/bco2.247

**Published:** 2023-05-19

**Authors:** Alfin Okullo, Jeremy Saad, Darius Ashrafi, Nasser Bagheri, Hodo Haxhimolla

**Affiliations:** ^1^ The Canberra Hospital Canberra Australian Capital Territory Australia; ^2^ The Australian National University Canberra Australian Capital Territory Australia; ^3^ National Capital Private Hospital Canberra Australian Capital Territory Australia

**Keywords:** bladder outlet obstruction, lower urinary tract symptoms, Millen's prostatectomy, retropubic prostatectomy, robot assisted

## Abstract

**Introduction:**

The study aims to demonstrate the feasibility, safety and efficacy of robotic simple prostatectomy (RSP) using the modified Freyer's approach in an Australian patient cohort. Although RSP is performed in several Australian centres, there is a paucity of published Australian data.

**Methods:**

We reviewed prospectively collected perioperative and outcomes data for patients who underwent a robotic modified Freyer's prostatectomy (RMFP) from June 2019 to March 2022. Statistics were completed using SPSS statistics v27.0 and reported as mean and range with a *p* value of <0.05 considered statistically significant.

**Results:**

There were 27 patients who underwent RMFP over the study period with a mean age of 67 years and prostate volume of 159.74 cc (100–275). The mean console time was 168 min (122–211), blood loss of 233 ml (50–600) and average length of hospital stay of 3.8 days (3–8). The preoperative versus postoperative outcome means were as follows: serum prostate‐specific antigen was 9.69 versus 1.2 ng/mL, IPPS score was 17.1 versus 1.25, quality of life (QOL) score 3.4 versus 0.4, postvoid residual volume: 223.6 versus 55.9 ml, Q‐max 7.86 versus 29.6 ml/s. These were all statistically significant (*p* < 0.001). The mean weight of resected tissue was 74 g (43–206) with 25 patients having benign histopathology and two being diagnosed with prostate cancer (Gleason 3 + 3 = 6 and 3 + 4 = 7). No patients returned to theatre or required a blood transfusion.

**Conclusions:**

Data from our patient cohort demonstrate the feasibility, safety and efficacy of RMFP for benign prostatic hyperplasia in an Australian patient cohort. Our outcomes compare favourably with published studies on RSP.

## 1 INTRODUCTION

Over the past two decades, surgical options available to treat men with symptomatic benign enlargement of the prostate have increased. Traditionally, transurethral resection of the prostate (TURP) has been the definitive option of choice.^1^ However, it is generally not recommended for large glands (>80 cc) on account of its morbidity with alternative options including laser enucleation.^2^ Despite this, open prostatectomy has remained a relatively popular option with a slew of differing approaches as described by Eugene Fuller (1884), Peter Freyer (1900) and Millin in 1947.^3^, ^4^, ^5^ They that noted for patients with a prostate gland of over 100 cc, large amounts of prostatic tissue could be removed with excellent and durable long‐term functional outcomes.^6^, ^7^

Open simple prostatectomy (OSP), however, remains a procedure with high morbidity due to the high transfusion rate and increased length of hospital stay.^8^ The laparoscopic approach slightly mitigated some of these drawbacks with comparable outcomes to OSP, but the complications rates remained concerningly high. This, combined with a technically challenging procedure and steep learning curve, has resulted in its low adaptation by urological surgeons.^9^, ^10^

The advent of robotic simple prostatectomy (RSP) over the past two decades has seen a significant reduction in complication rates associated with simple prostatectomy. The robot allows for better magnification, 3D vision, stability, superior ergonomics and dexterity, facilitating completion of the more challenging aspects of the OSP as demonstrated in international studies.^11^ Although several Australian centres are currently performing the RSP, albeit overwhelmingly within the private healthcare system, there are no published local outcomes data.

With this study, we aimed to ascertain the feasibility, safety and efficacy of RSP in an Australian patient cohort using a modified Freyer's approach.

## 2 METHODS

### 2.1 Study cohort

Twenty‐seven patients with persistent symptoms and complications related to benign prostatic enlargement (BPE) underwent a robotic modified Freyer's prostatectomy (RMFP) between June 2019 and March 2022. The clinical work‐up included a history, International Prostate Symptom Score (IPSS) at the initial clinical consultation and physical examination. Serum prostate‐specific antigen (PSA) levels were checked, and a renal tract ultrasound scan and flow tests were completed.

All patients with a concerning PSA level and or digital rectal exam findings underwent further work‐up for prostate cancer including repeat serum PSA level checks, prostate MRI and biopsy as indicated. All patients had undergone a period of medical therapy prior to consideration for surgery on either tamsulosin or tamsulosin and dutasteride combination (Duodart). Other surgical options including bipolar TURP, LASER, photo‐vaporization and open prostatectomy were discussed during the consent process.

### 2.2 Equipment and positioning

RMFP was performed using the da Vinci Xi Surgical system (Intuitive Surgical, Sunnyvale, CA, USA). Patients were positioned supine with 18° Trendelenburg. Five transperitoneal ports are placed akin to the robotic radical prostatectomy port placement.

### 2.3 Surgical technique

**FIGURE 1:**
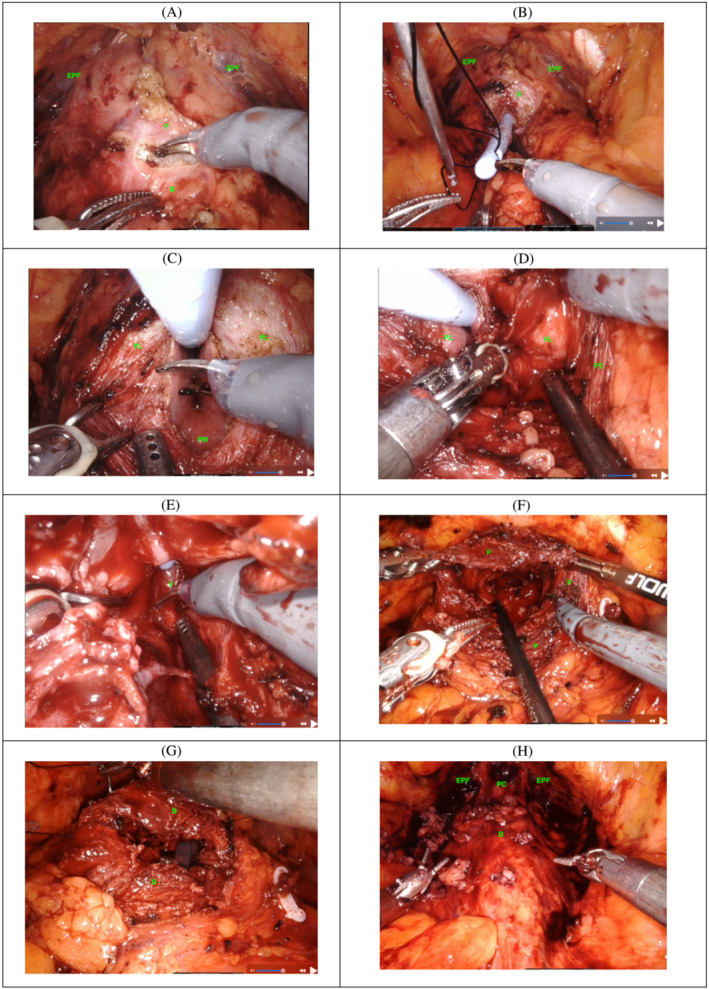
(A–F) Technique of robotic modified Freyer's prostatectomy. B, bladder; EPF, endopelvic fascia; P, prostate; PC, prostate capsule; PL, prostate lateral lobe; PM, prostate median lobe; V, verumontanum.

The bladder neck is identified, a transverse incision is then made through the prostate capsule just caudal to the prostato‐vesical junction anteriorly between the 7 and 5 o'clock positions (Figure 1A). The IDC is grasped and used to traction the prostate anteriorly. (Figure 1B) The plane between the adenoma and the prostate capsule is developed using sharp and blunt dissection starting with the lateral lobes. (Figure 1C,D) At the apex, the catheter is withdrawn until the verumontanum is identified. An incision is made cranial to the verumontanum. (Figure 1E) Apical dissection and enucleation is performed to leave only the prostatic capsule with all adenoma removed (Figure 1F).

Meticulous haemostasias is then performed with 3.0 PDS sutures. Only minimal cautery is employed. The enucleated tissue is placed in an end‐o‐catch bag. The cystotomy is closed with 2‐0 V‐Loc sutures (Covidien, Norwalk, CT, USA) (Figure 1E,F) over a 24Fr IDC with 40–60 ml of water in the balloon. The bladder is flushed with saline to ensure any significant clot is extracted and a leak test performed. Extra sutures are applied to any identified leaking points. A 19fr Blake drain is inserted in the rectovesical pouch. The robot is then un‐docked, and the specimen is extracted through the robotic assistant port‐site.

### 2.4 Postoperative care

Intravenous antibiotics were continued for 24 h postoperatively. Continuous bladder irrigation was commenced. Deep venous thrombosis prophylaxis with subcutaneous heparin was commenced in the immediate postoperative period. Drain outputs were monitored and recorded. Drains were removed typically on postoperative Day 2, when the outputs were deemed insignificant (less than 30 ml over a 24‐h period). Patients were discharged with the indwelling catheter in‐situ. A follow‐up cystogram was arranged and reviewed by the urologist to determine when it was appropriate to remove the catheter.

### 2.5 Data collection

A retrospective review of prospectively collected data was performed. Patient demographics included patient age, preoperative symptoms and flow tests, medications for BPE, imaging studies to determine prostate size, preoperative postvoid residual volumes (PVRs) and PSA levels. Operative data included American Society of Anesthesiologists (ASA) score, console time and estimated blood loss. Postoperative data included length of stay, catheter dwell time and postoperative complications. Outcomes data included weight and histopathological assessment of enucleated tissue. Postoperative functional outcome data included postoperative PVRs, quality of life (QOL) scores and Q‐max measurements.

Approval to carry out this study was granted by the ACT Health Human Research Ethics Committee (Approval number 2022/ETH00035).

### 2.6 Statistical methods

We used SPSS statistics v27.0 (IBM Corp, NY, USA) software for statistical analysis. Continuous variables were recorded as mean and range with categorical variables expressed as a percentage. Statistical significance was determined using a two‐sided significance level of 0.05 calculated using the paired *t*‐test to compare means.

## 3 RESULTS

We enrolled 27 patients who underwent a RMFP between June 2019 and March 2022. All the patients were operated on at the same hospital and by a single surgeon. Table 1 presents patients demographics and clinical characteristics of the included patients. The mean patient age was 67.5 years (range 55–75). All patients had undergone a period of unsuccessful medical management with a mean duration of medical management of 18 months (range 13–22). The mean ASA score was 2.38 (range 2–3), and the mean body mass index was 28.5 (range 20.8–39.9).

**TABLE 1:** Patient demographics and preoperative parameters.

Patient demographics	Results
Age, year, mean (range)	67.5 (55–75)
BMI, kg/m^2^, mean (range)	28.59 (20.8–39.9)
Intravesical median lobe	26 (96.3%)
ASA, mean (range)	2.38 (2–3)
IPSS score, mean (range)	17.1 (3–35)
AUA‐QOL score (range)	3.5 (0–6)
Q‐max, ml/s, mean (range)	7.86 (2.8–17.4)
PSA, ng/ml, mean (range)	9.69 (1.3–51)
Prostate volume, ml, mean (range)	159.74 (88–275)
Postvoid residual, ml, mean (range)	223.6 (30–615)
BPH‐related complications, no (%)	
Bladder calculi	3 (11.1%)
Urinary retention	6 (22.2%)
Catheter dependent	3 (11.1%)
Bladder diverticulum	1 (3.7%)
Urinary tract infection	9 (33.35)
Visible haematuria	8 (29.6%)

Abbreviations: ASA, American Society of Anesthesiologists; AUA‐QOL, American Urological Society Quality of Life score; BMI, body mass index; BPH, benign prostatic hyperplasia; IPSS, International Prostate Symptom Score; PSA, prostate‐specific antigen.

### 3.1 Preoperative parameters

Table 2 presents preoperative date. The mean preop prostate volume was 159.74 (range 88–275), with a mean PSA of 9.69 (range 1.3–51). The mean American Urological Association (AUA)‐IPSS score was 17.1 (range 3–35) and AUA‐QOL score was 3.5 (range 0–6). The mean PVR was 223.6 (range 30–615) with a Q‐max of 7.86 (range 2.8–17.4).

**TABLE 2:** Peri‐operative data.

Parameter	Results
Console time, min, mean (range)	168 (122–211)
Estimated blood loss, ml, mean (range)	233 (50–600)
Concomitant procedures, no, (%)	1(3.7%)
Postoperative blood transfusions, no (%)	0 (0%)
Conversions, no, (%)	0(0%)
Preop haemoglobin, g/dL, mean (range)	147 (127–174)
Postoperative haemoglobin, g/dL, mean (range)	129 (117–156)
Weight of prostate tissue resected, grammes, mean (range)	74 (43–206)
Length of hospital stay, days, mean (range)	3.83 (3–8)
Pelvic drain dwell time, day, mean (range)	1.9 (1–4)
Catheter dwell time, days, mean (range)	6.68 (4–8)
Postoperative serum PSA, ng/dL, mean (range)	1.2 (0.08–4.9)
Clavien–Dindo complications, no (%)	
Grade 1	5 (18.0%)
Grade 2	0 (0%)
Grade 3a	0 (0%)
Grade 3b	0 (0%)
Grade 4 or 5	0 (0%)

Abbreviation: PSA, prostate‐specific antigen.

### 3.2 Peri‐operative parameters and complications

The mean operating duration was 168 minutes (range 122–211) with an estimated blood loss of 233ml (range 50–600). There were no cases that required conversion during the RMP and no significant intra‐operative complications. The average length of hospital stay was 3.83 days (range 3–8 days) with an average drain and catheter dwell time of 1.9 days (range 1–4) and 6.68 days (range 4–8 days), respectively. Trial of void was performed on postoperative day (POD) 4 for 1 patient, POD 5 for 2 patients, POD 6 for 10 patients, POD 7 for 13 patients and on POD 8 for 1 patient.

Three patients were reported as having a ‘small leak’ on their POD 7 cystograms by the radiologists. On re‐review of the cystograms by the surgeon, these were deemed insignificant, and all three patients proceeded to a TOV with no adverse outcomes. One patient had a concomitant robotic cystolithotomy in addition to the RMP. There were four postoperative complications that however did not require a change in the usual management course. One patient had bleeding from the drain site, which was managed by application of pressure. No patients had bleeding complications after catheter removal requiring re‐catheterization or bladder manual washout‐out. There were no cases of urinary tract infections.

The mean weight of the resected specimen was 74 g (range 43–206). Two cases of prostate cancer were identified. One had a small focus of Gleason 6 (3 + 3 = 6) prostate cancer whereas the other had Gleason 7 (3 + 4 = 7) cancer. The former patient is on active surveillance, and the latter underwent radiotherapy for prostate cancer. Functional outcomes were assessed with a mean follow‐up period of 15.6 months (range 3–31 months). Mean postoperative IPSS score was 1.25 (range 0–6), representing a 92.7% improvement from preoperative IPSS scores (*p* < 0.05). The mean PVR was 55.9 ml (range 0–303), representing a 75% reduction from presurgery PVR with three patients having a PVR of greater than 100 ml. The mean postoperative flow rate increased to 29.6 ml/s (range 9.3–53) compared with 7.86 (range 2.8–17.4) preoperatively, representing a 73.4% improvement in Q‐max (*p* < 0.05).

The mean serum PSA levels decreased to 1.2 (range 0.08–4.9) from a preoperative mean of 9.69 (range 1.3–51), which represents an 87.6% drop in serum PSA (*p* < 0.05). None of the patients have reported incontinence following the RMP.

## 4 DISCUSSION

For men with moderate to severe LUTS and prostate sizes greater than 80 cc, the European Association of Urology and AUA guidelines recommend a simple prostatectomy performed either by an open, laparoscopic (LSP) or robotic approach where a transurethral bipolar enucleation or Holmium laser enucleation of the prostate (HoLEP) is not available.^12^, ^13^ OSP is more likely to be considered in glands larger than 100 cc. Although with improvements in the safety profile of newer technologies like photo‐vaporization of the prostate (PVP) and HoLEP, there is now a wider array of treatment options. The short‐term outcomes of these laser treatments have been compared with OSP for large glands with a demonstration of equivalent outcomes in improvement in IPSS, Q‐max and PVR.^14^, ^15^

A major drawback of OSP is the associated complication rates and in particular peri‐operative bleeding requiring blood transfusions. Gratzke et al.^16^ in their large series of 902 patients undergoing OSP reported an overall complication rate of 17% with 68 (7.5%) patients requiring blood transfusion and 33 (3.7%) of patients requiring surgical revision for severe bleeding. They concluded that although OSP offered satisfactory short‐term outcomes, it should probably be offered where surgeons have no access to, or training in LSP or modern technologies like HoLEP or PVP.

Furthermore, in a study by Kuntz and Lehrich in which they randomized patients between HoLEP and OSP for glands greater than 100 cc, they found a higher complication rate with OSP versus HoLEP (26.7% vs. 15%) with a 13.3% transfusion rate for patients undergoing OSP.^15^

Some of the shortcomings of OSP include a longer recovery time albeit partly mitigated by the advent of LSP. Mariano et al.^9^ found that LSP results in less postoperative pain and a quicker recovery. However, it was associated with a higher transfusion rate of 15.8% compared with 10.8% in the OSP group in a study by McCullough et al.^10^ This is almost certainly related to the physically demanding ergonomics of laparoscopic surgery within the confines of the pelvis but also the limited visualization and challenges in performing intracorporeal suturing to control bleeding vessels. This would largely explain the subsequent poor uptake of LSP as an improvement on OSP. With the advent of robotic surgery and its popularity in urology, most of the challenges arising from OSP and LSP have been overcome.

Currently, HoLEP is also considered a reasonable option to RSP although a few differences should be borne in mind. The learning curve for RSP has been reported at approximately 10–12 cases.^17^ Robotic surgeons with pelvic surgical experience are already familiar with the anatomy further shortening the learning curve. In contrast, Brunckhorst et al.^18^ found the learning curve for HoLEP to be about 50 cases with a high complication rate of approximately 20% for the first 40 cases. Robert et al.^19^ reported the learning curve for HoLEP as a minimum of 20 cases with improved outcomes after 50 cases.

HoLEP requires the use of a 26F sheath, which not infrequently requires urethral dilatation and its associated complications. In addition, further challenges are encountered in extracting the enucleated adenoma increasing the risk of urethral injury and stricture. For patients undergoing a second procedure, the urethral stricture rate following TURP has been reported at approximately 6.5% and 3.3% in HoLEP.^20^, ^21^ In comparison, because RSP is not performed transurethrally, there has been no strictures as yet reported.

Transient incontinence following HoLEP is also a concern. According to Cho et al.,^22^ 4.6% (18 of 393) of patients who underwent HoLEP experienced urinary incontinence for longer than 3 months. Shigemura et al.^23^ reported the outcomes of 203 HoLEP procedures and found postoperative stress urinary incontinence (SUI) was reported at 1, 3 and 6 months in 35 (29.4%), 20 (16.8%), and 6 (5.04%) patients, respectively, who underwent surgery by trainee surgeons and in 32 (38.1%), 11 (13.1%) and 4 patients (4.76%), respectively, who were treated by experienced surgeons. About 5% of the patients in this study reported urinary incontinence lasting more than 6 months. Das et al.^24^ reported even much higher transient postoperative SUI rates of up to 44%.

No urinary incontinence was reported in our patient cohort and in other RSP series. This can be attributed to the fact RSP avoids the transurethral approach and no dissection is carried out distal to the verumontanum hence avoiding injury to the external sphincter.

Robotic surgery enables surgeons to simulate OSP but with vastly superior manual dexterity, visualization and access to the pelvis and prostate cavity. This allows for a more precise anatomic enucleation of the adenoma and easier intracorporeal suturing. In comparison with pure LSP, it shows comparable outcomes but portends less surgeon fatigue with better access and dexterity.^3^, ^25^

The EBL of 233 ml (range 50–600 ml) in our study with none of the patients requiring a blood transfusion compares favourably with figures from the published international studies on RSP (Table 3). An outlier series by Sotelo et al.^30^ reported a 14% transfusion rate.^30^ However, it is the earliest published series at a time when the technique was almost certainly still in its infancy. As has been described by Leslie et al.,^28^ several factors contribute to the lower transfusion rates in RSP but key among them is the tamponading effect of the pneumoperitoneum on open venous channels within the prostatic fossa. Others include superior 3D vision and articulation of the robotic instruments allowing for access to and control of bleeding vessels either by diathermy or suture ligation irrespective of their location at the bladder neck or prostatic apex.

**TABLE 3:** Summary of published robotic simple prostatectomy series.

Author name	Number of patients	Mean age (years)	Mean preoperative IPSS	Mean operative time (min)	Mean operative blood loss (ml)	Mean resected prostate (g)	Catheterisation time (days)	Median preoperative Q‐max (ml/s)	Median postoperative Q‐max (ml/s)	Mean postoperative IPSS
Sotelo et al., 2008^24^	7	64.7	20.9	195	382	50.5	7	17.7	55.5	7.5
Yuh et al., 2008^26^	3	76.7	17.7	211	558	301	NR	NR	NR	NR
John et al., 2009^27^	13	70	NR	210	500	82	6	NR	23	NR
Uffort and Jensen, 2010^28^	15	65.8	23.9	128.8	139.3	46.4	4.6	NR	NR	8.13
Sutherland et al., 2011^29^	9	68	17.8	183	206	112	13	NR	NR	7.8
Vora et al., 2012^30^	13	67.1	18.2	179	219	163.8	8.8	4.37	19.1	5.3
Matei et al., 2012^31^	35	65.2	24	186	121	87	7.4	7.5	18.3	5
Falavolti et al., 2017^32^	18	74	8	205	200	100	5.6	NR	NR	25.2
Banapour et al., 2014^33^	16	68.4	22	228	197	94.2	8	NR	NR	7
Leslie et al., 2014^25^	25	72.9	23.9	214	143	88	9	11.3	20	3.58
Pokorny et al., 2015^34^	67	69	25	97	200	84	3	7	23	3
Wang et al., 2016^35^	10	68	25.1	150	100	79	12	9.9	24.5	NR
Mourmouris et al., 2019^36^	26	66.7	22.87	134	274	115.3	3	10.11	19.12	5.7
R.Dotzauer et al., 2021^37^	103	71	17.3	182	248	NR	6	6.1	NR	NR
Present series, 2021	13	67.8	20.5	107	92.5	82.8	5	7	15.1	4.9

The preoperative use of dutasteride for at least 6 weeks has also been noted to reduce intra‐operative blood loss in a study by Gokce et al.^29^ Bleeding risk in these patients may also be exacerbated by a large median lobe, which interferes with exposure and makes suturing difficult. An intravesical median lobe was present in 96% (26/27) of our patients. Improved visualization, gas compression of the venous system and a stitch for traction can be used to reduce intraoperative blood loss.^38^, ^39^ We also managed bleeding of the prostatic fossa with 3‐0 V‐Loc continuous sutures, which has been used effectively in other studies.^31^

One of the generally accepted benefits of robotic surgery is the significantly decreased length of hospitalization compared to an open procedure. Our mean hospital stay was 3.83 days (range 3–8), which is comparable with the published literature. A few series have reported even shorter mean length of stay of between 1 and 2.7 days.^26^, ^27^, ^30^, ^32^, ^33^

We had only a few postoperative complications (18%) with one patient bleeding from the drain site and three patient's cystograms reported as having a small leak. These were all Grade 1 Clavien–Dindo complications. There were no urinary tract infections in our patient cohort despite a mean catheterization period of 6.68 days (range 4–8). This might have arisen from the practice of administering intravenous antibiotics for 24 h following a RMP for all our patients.

The functional outcomes in our patient cohort were excellent and found to be in keeping with the trend in comparable studies (Table 4). Our outcomes were assessed at a mean of 15 months postoperative (range 3–31). Long‐term data on functional outcomes following RSP are still lacking, but because the adenoma enucleated is comparable with OSP, the same outcomes could be extrapolated to RSP with low re‐operation rates of 2%–5% reported.^6^, ^7^

**TABLE 4:** Functional outcomes of robotic modified Freyer's prostatectomy.

Parameter	Preoperative mean (range)	Postoperative mean (range)	Percent change	*p* value
IPSS score.	17.1 (3–35)	1.25 (0–6)	−92.7	<0.0001
AUA‐QOL score	3.4 (0–6)	0.4 (0–2)	−88.2	<0.0001
Q‐max, ml/s	7.86 (2.8–17.4)	29.6 (9.3–53)	+73.4	<0.0001
PSA, ng/ml	9.69 (1.3–51)	1.2 (0.1–4.9)	−87.6	<0.0001
PVR, ml	223.6 (30–615)	55.9 (0–303)	−75	<0.0001

Abbreviations: AUA‐QOL score, American Urological Society Quality of Life score; IPSS, International Prostate Symptom Score; PSA, prostate‐specific antigen; PVR, postvoid residual; Q‐max, maximum flow rate.

The amount of prostate tissue enucleated is an indicator of the potential durability of the procedure as has been demonstrated in a prospective randomized trial comparing PVP and OSP. Postoperative prostate volume at 18 months had decreased less in the PVP cohort with poorer IPSS QOL scores at 18 months.^34^ The mean weight of enucleated tissue in our patient cohort was 74 g (range 43–206). Comparable studies with similarly excellent functional outcomes have reported resected mean tissue weights between 46 and 163.8 g (Table 3). A mean postoperative PSA drop of 87.6% also alludes to the completeness of prostate enucleation using the robotic approach.

The prior robotic surgical experience of the primary surgeon in our series includes over 3000 robotic procedures. This, as already mentioned, helped shorten the learning curve for a RSP.

The primary surgeon used a modification of the Freyer approach by incising the bladder at the bladder neck. A few advantages of the RMFP over the traditional Millin and Freyer approaches are as follows

- It affords a more familiar dissection for surgeons already competent in robotic assisted radical prostatectomy (RARP) because the bladder incision for the RMFP is the same as for the RARP.
- A smaller incision is made at the anterior bladder neck as compared with the wider incision at the posterior bladder wall with the traditional Freyer approach. This should cause less scarring to the bladder.
- Direct visualization of the ureteric orifices potentially affords a greater level of safety than that achieved in the Millin's approach.
- For surgeons still early in the learning curve, RMFP offers a potentially easier enucleation of the adenoma from the capsule compared with the Millin's approach with less likelihood of straying off the dissection plane compared to the Millin's approach.
- Median lobe access is enhanced through the RMFP approach, hence facilitating its dissection.
- A drawback of the RMFP would be a potentially higher morbidity of the procedure as compared with the Freyer approach because the space of Retzius has to be dissected.

With the increasing access to robotic procedures in many public and private hospitals in Australia, the RMFP procedure would be a useful addition to the options for patients presenting with symptoms from moderate to large sized glands.

### 4.1 Limitations

Our study size is limited to 27 patients, hence not adequately powered to enable reproducibility. Larger studies would go a long way in galvanizing evidence of the utility and efficacy of this procedure. Our mean follow‐up period was 15 months and was not adequate to review long‐term functional outcomes with desirable longer follow‐up.

This is a single‐surgeon series for a surgeon with vast experience in robotic surgery, and therefore, the outcomes might not be similar for surgeons with lesser experience.

Finally, the question of cost was not addressed in the current study. Matei et al.^35^ have however demonstrated that although the initial operative costs were higher for RSP, the overall hospitalization costs were lower when compared with both OSP and TURP.

## 5 CONCLUSIONS

Perioperative and functional outcomes data from our study demonstrate the safety, feasibility and efficacy of the RMP in Australian patients with large and symptomatic prostatic hypertrophy. Although our study cohort is drawn from the private healthcare system, with the increasing availability of the surgical robot in the Australian public healthcare system, it is hoped that this study will act as a reference for the provision of this procedure to all Australian men.

## AUTHOR CONTRIBUTIONS

**Alfin Okullo:** Writing—original draft; conceptualization. **Jeremy Saad:** Writing—review and editing. **Darius Ashrafi:** Writing—review and editing. **Nasser Bagheri:** Formal analysis; resources. **Hodo Haxhimolla:** Conceptualization; supervision.

## CONFLICT OF INTEREST STATEMENT

The authors declare no conflicts of interest.
